# Impaired receptivity of thin endometrium: therapeutic potential of mesenchymal stem cells

**DOI:** 10.3389/fendo.2023.1268990

**Published:** 2024-01-25

**Authors:** Michael H. Saad-Naguib, Yannick Kenfack, Lauren S. Sherman, Olivia B. Chafitz, Sara S. Morelli

**Affiliations:** ^1^ Department of Obstetrics, Gynecology & Reproductive Health, Rutgers Biomedical and Health Sciences, Rutgers, The State University of New Jersey, Newark, NJ, United States; ^2^ Department of Medicine, Rutgers Biomedical and Health Sciences, Rutgers, The State University of New Jersey, Newark, NJ, United States; ^3^ Department of Obstetrics & Gynecology, Hackensack University Medical Center, Hackensack, NJ, United States

**Keywords:** mesenchymal stem cells, thin endometrium, Asherman’s syndrome, endometrial regeneration, endometrial receptivity

## Abstract

The endometrium is a resilient and highly dynamic tissue, undergoing cyclic renewal in preparation for embryo implantation. Cyclic endometrial regeneration depends on the intact function of several cell types, including parenchymal, endothelial, and immune cells, as well as adult stem cells that can arise from endometrial or extrauterine sources. The ability of the endometrium to undergo rapid, repeated regeneration without scarring is unique to this tissue. However, if this tissue renewal process is disrupted or dysfunctional, women may present clinically with infertility due to endometrial scarring or persistent atrophic/thin endometrium. Such disorders are rate-limiting in the treatment of female infertility and in the success of *in vitro* fertilization because of a dearth of treatment options specifically targeting the endometrium. A growing number of studies have explored the potential of adult stem cells, including mesenchymal stem cells (MSCs), to treat women with disorders of endometrial regeneration. MSCs are multipotent adult stem cells with capacity to differentiate into cells such as adipocytes, chondrocytes, and osteoblasts. In addition to their differentiation capacity, MSCs migrate toward injured sites where they secrete bioactive factors (e.g. cytokines, chemokines, growth factors, proteins and extracellular vesicles) to aid in tissue repair. These factors modulate biological processes critical for tissue regeneration, such as angiogenesis, cell migration and immunomodulation. The MSC secretome has therefore attracted significant attention for its therapeutic potential. In the uterus, studies utilizing rodent models and limited human trials have shown a potential benefit of MSCs and the MSC secretome in treatment of endometrial infertility. This review will explore the potential of MSCs to treat women with impaired endometrial receptivity due to a thin endometrium or endometrial scarring. We will provide context supporting leveraging MSCs for this purpose by including a review of mechanisms by which the MSC secretome promotes regeneration and repair of nonreproductive tissues.

## 1 Introduction

The endometrium is a highly dynamic tissue, undergoing ~400 repeated cycles of proliferation, differentiation, shedding, and regeneration over a woman’s reproductive lifespan. Cyclic regeneration of the endometrium is a prerequisite for embryo implantation and requires hormonal stimulation, inflammation, angiogenesis, and reepithelization of the uterine lining following menses (1). The ability of the endometrium to undergo rapid, repeated regeneration during the proliferative phase, without scarring, is unique to this tissue and is an absolute requirement for the establishment of a human pregnancy. The secretory phase of the endometrial cycle commences after ovulation, during which time local inflammatory events involving immune cells, cytokines, and chemokines play a crucial role in decidualization of the endometrium in preparation for implantation (2).

Embryo implantation is defined as the process by which a blastocyst adheres to and invades the endometrium, prompting development of the feto-maternal interface (3). This process requires a complex crosstalk between a competent embryo and a receptive endometrium. Endometrial receptivity is acquired during a specific time frame post-ovulation in the menstrual cycle, when genotypic and phenotypic changes in endometrial cells result in a conducive environment for blastocyst implantation. This timeframe, known as the “window of implantation”, is also marked by cellular expression of various cytokines, growth factors, and prostaglandins necessary for synchronized crosstalk between the blastocyst and endometrium (3, 4). Beyond this timeframe, the endometrium is considered refractory to implantation or “nonreceptive”, during which time the embryo cannot establish contact, and successful implantation will not occur (4).

Despite significant advancements in assisted reproductive technologies (ART) over the past few decades, embryo implantation remains a rate limiting step in achieving success after *in vitro* fertilization (IVF). In modern practice, where high quality (and often euploid) embryos are identified and selectively transferred, impaired endometrial receptivity may contribute to as many as two thirds of embryo implantation failures (5). Impaired regeneration of the endometrium predisposes to a thin endometrium, which is an important contributor to impaired endometrial receptivity during IVF cycles (6). Based on a meta-analysis of 22 studies, 2.4% of women undergoing IVF are affected by thin endometrium, typically defined as an endometrial thickness less than 7 mm on transvaginal ultrasound (7). Asherman’s syndrome is one common cause of impaired endometrial regeneration and thin endometrium, and is characterized by intrauterine adhesions causing menstrual abnormalities, infertility, and/or recurrent pregnancy loss (8). The prevalence of Asherman’s syndrome varies drastically by subpopulation, ranging from 2.8% to 45.5% among infertile women (9). Most cases result from trauma to the gravid uterus, with significantly fewer cases resulting from trauma to the nongravid uterus or genital infections such as tuberculosis (10). The inflammation and fibrosis caused by these insults impairs regeneration of the endometrial lining. Thin endometrium due to any cause also increases with age, with an incidence as high as 25% of women undergoing IVF over age 40 (11).

Regardless of the etiology of thin endometrium, it is well established that embryo transfers performed in the setting of thin endometrium have decreased implantation and clinical pregnancy rates (12). A 2014 meta-analysis found that women with an endometrial thickness < 7.0mm had lower clinical pregnancy rates than women with an endometrial thickness of 7.0mm and above (7). More specifically, a retrospective cohort analysis of more than 40,000 embryo transfers found that clinical pregnancy and live birth rates decreased with each millimeter decline in endometrial thickness below 8 mm in fresh IVF-embryo transfer cycles, and below 7 mm in frozen-thawed embryo transfer cycles (13). In women who conceive, these pregnancies are subject to impaired placentation (14) and associated pregnancy complications, including early spontaneous miscarriage (15), preterm birth (16, 17), low birth weight (16, 17), hypertensive disorders of pregnancy (16, 18), placental abruption (16) and ectopic pregnancy (15).

Despite the scope of the clinical problem, therapeutic options for thin endometrium remain extremely limited. Numerous therapeutic options have been evaluated for thin endometrium; multiple medication regimens including high dose estradiol, vitamin E and pentoxifylline, mid-luteal GnRH agonist, tamoxifen, and sildenafil have been investigated (19), with modest and varying degrees of success. Immunomodulatory or pro-inflammatory methods, such as endometrial scratching (20), stem cell transplant, and intrauterine granulocyte-colony stimulating factor (G-CSF) instillation have also been employed (19). However, despite the profound limitations that thin endometrium imposes on ART and pregnancy outcomes, the optimal therapy for this condition has not yet been established.

A growing number of studies have explored the potential of mesenchymal stem cells (MSCs), a specific adult stem cell type, to treat women with disorders of endometrial regeneration. MSCs are multipotent adult stem cells with capacity to differentiate into cells of multiple lineages (21, 22). In addition to their differentiation capacity, MSCs migrate toward injured sites where they secrete bioactive factors that modulate biological processes critical for tissue regeneration (23–25). The MSC secretome has therefore attracted significant attention for its therapeutic potential in regenerative medicine, including in the uterus. Our objective herein is to provide a brief review of MSC biology and explore the potential of MSCs to treat women with impaired endometrial regeneration, based on mechanisms elucidated via a growing number of studies in rodent and human models.

## 2 Mesenchymal stem cells

**Figure 1:**
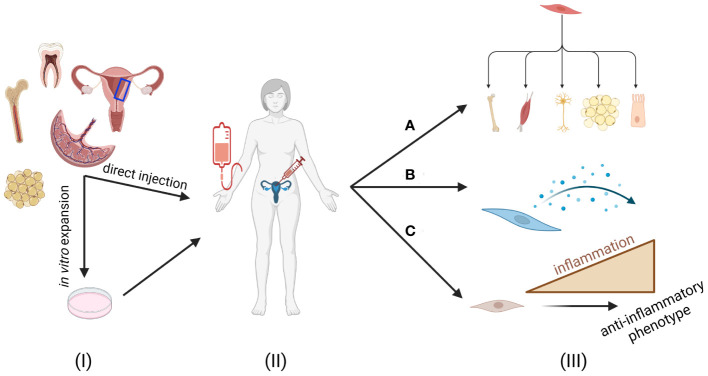
MSCs for treatment of endometrial disorders. (I) Mesenchymal stem cells (MSCs) can be isolated from various adult tissues, including bone marrow, adipose, placenta, umbilical cord, dental pulp, endometrial tissue, and menstrual blood for autologous use. These cells can be directly administered to a patient with or without minimal modification or can be expanded *in vitro* prior to administration. (II) Once ready for injection, the MSCs can be introduced systemically (i.e. intravenously) or locally to exert their responses. (III) MSC effects can be due to a combination of their **(A)** differentiation potential, **(B)** secretome, and **(C)** licensing to an anti-inflammatory phenotype. These functions in conjunction make MSCs an attractive candidate for treating endometrial disorders. Figure created with Biorender.com.

Mesenchymal stem cells (MSCs), alternatively known as mesenchymal stromal cells, are multi-lineage, non-hematopoietic stem cells with self-renewing properties. First discovered by Alexander Friedenstein in the 1960s, MSCs are found throughout multiple adult and fetal tissues including bone marrow, adipose, dental pulp, umbilical cord, placenta, endometrial tissue, and menstrual blood (21, 26, 27). MSCs from all tissue sources are characterized by the expression of CD29, CD44, CD73, CD105, and CD166; while lacking hematopoietic stem cell markers such as CD34 and CD45 (23). MSCs generate various connective tissues including bone, fat, muscle, and cartilage; and have been found to generate various other functional cell types including myocytes and varying subsets of neurons (21, 22). Beyond their capacity to differentiate into various cell types, MSCs are associated with multiple endocrine and paracrine functions, including angiogenesis, homing to areas of inflammation, and systemic modulation of immune responses – both pro- and anti-inflammatory in nature, depending on the microenvironment – via their secretome, including soluble factors, neuropeptides, and microvesicles (**Figure 1**) (23–25). Critically, MSCs can cross allogeneic barriers, permitting MSCs derived from any patient to be used as an off-the-shelf technology for third party recipients (25).

Isolation of MSCs for expansion or direct use can be accomplished through minimally invasive procedures including bone marrow aspiration, abdominoplasty, lipoaspiration, and collections of other discard tissues such as the placenta and menstrual blood (28, 29). From here, the cells can be used as a heterogeneous population or can be enriched for specific population(s) of MSCs. Together with their ease of isolation and capacity to cross allogeneic barriers, this further reduces safety and ethical concerns toward MSC use, adding to the MSCs’ clinical appeal.

To date MSCs are listed in use in over two thousand clinical trials (clinicaltrials.gov), capitalizing on the aforementioned properties of MSCs – primarily their ability to home to areas of inflammation, reduce inflammation locally and/or systemically, and promote tissue regeneration (23, 25, 30–33). Beyond these currently listed applications, MSCs are being studied as potential therapy in conditions such as cancer, diabetes mellitus, and end-stage liver disease. In the subsequent sections we will discuss how MSCs modulate the immune system’s inflammatory response and promote tissue regeneration, and we will also discuss emerging uses of MSCs in endometrial dysfunction.

### 2.1 Homing of MSCs

Migration of MSCs from either their microenvironmental niche or site of administration to the target tissue or organ is a fundamental characteristic of MSCs that facilitates their response to inflammation and tissue regeneration. MSC homing, which refers to MSC migration toward a target tissue, is facilitated by an array of cytokines and chemokines released at the site of inflammation (23, 28). MSCs express a wide array of cytokine and chemokine receptors permitting their migration and response to rapidly changing microenvironments (34, 35). Of note, MSCs express CXCL12, the receptor for SDF1α, permitting their chemo-attraction to sites of inflammation (36). Beyond SDF1α, other yet undefined cytokines are believed to be part of this chemoattraction; these cytokines could therefore be targeted for direct therapy, such as in the case of immune regulation, which will be discussed below, or as a mechanism to deliver another therapy, e.g., drug delivery (37). Once at their site, MSCs can be directly involved in tissue regeneration, but MSCs predominantly exert their function via their secretome (24, 25, 38). The efficiency of exogenous MSC mobilization to the target tissue significantly impacts the therapeutic efficacy of these cells.

Despite its importance, MSC mobilization is very inefficient (<10%), leading to debate over optimal routes of administration to improve mobilization for different applications (38). Local administration of MSCs to the target organ or tissue is one route that may enhance MSC effect. However, this mode of delivery of MSCs can be highly invasive, and the MSCs often escape from the site of administration (39). Intravenous administration has also been suggested; however, most of the cells administered intravenously are entrapped in the lung due to the MSCs’ large size and their expression of adhesion molecules such as VCAM1 and VLA-4 which can bind lung epithelial cells, with estimates of only 1% of the cells reaching their target organ (40–42). The half-life of these entrapped MSCs is short – under four days – supporting the role of the MSC secretome in the observed clinical effects of MSCs (43–45).

Different molecular engineering approaches have been employed to increase MSC homing to specific sites, including overexpression of cytokine receptors and downregulation of adhesion molecules (46–51). Another approach shown to enhance MSC homing is magnetic guidance to the target tissue, whereby the MSCs are labeled with magnetic carbon nanotubules prior to administration (52). Notably these studies have largely found decreased lung entrapment and increased homing of MSCs to the target tissues without significant effect on the viability of the cells or their capacity to differentiate (48, 52). It is crucial to keep in mind that when it comes to enhancing the homing of MSCs and their response, the approach will differ depending on the target organ or tissue, the specific disease being treated, and the source and pre-treatment of the MSCs: understanding these nuances is key to developing effective treatments and improving patient outcomes.

### 2.2 MSCs as endocrine and paracrine mediators

As described above, there is little evidence to attribute the basis of MSC clinical effect to their tissue engraftment: only a small percentage of MSCs reach their target organ, and even this small population does not survive long-term (24). Thus, the primary mechanism of the MSCs is likely their release of soluble factors, including cytokines and microvesicles (MVs).

Among classes of MVs, exosomes are the most investigated. Exosomes are MVs of 30-150 nm in diameter, enriched in proteins, miRNA, lipids, and other regulatory molecules; they have been identified in a wide array of body fluids such as blood (including plasma and serum), urine, saliva, cerebrospinal fluid, pleural effusion, and ascites; and are considered representative of the cells from which they were derived [discussed in (53)]. Due to their source-cell representation, circulating MVs are under investigation for diagnostic purposes, evaluating for biomarkers for the presence and type of aberrant cells – including malignancy, injury, and other disorders (53–56). It thus stands to reason that the MVs derived from MSCs of different source tissues, and different treatments and exposures *in vitro*, would differ from one another (57, 58). Due to their similarity in effect with MSCs themselves, MSC-derived exosomes (MSC-Exos) are under clinical investigation for treatment of similar pathologies (59).

MSC-Exos have been associated with anti-inflammatory and reparative functions (58, 60–63). Over 2000 proteins have been identified within MSC-Exos (57, 58). These include cytokines (e.g. IL-10, IL-6, TGF-β, TNF-α), chemokines (e.g. MCP1, CXCL14, MCP3, SDF-1α), and trophic factors (e.g. FGF, HGF, IGF1, VEGF) (58). Like their parent cells, MSC-Exos can suppress effector T cells, dendritic cell (DC) maturation, M1 macrophages, and natural killer cells (NKs); while enhancing regulatory T cells and M2 macrophages (59, 64–66). Similarly, these cargo contribute to MSC-Exos regenerative capacity, enhancing osteogenesis, chondrogenesis, and angiogenesis (63, 67–69). It should be noted, however, that both direct and indirect forms of intercellular communication between MSCs and target cells have been reported: for example gap junctional intercellular communication (GJIC) for direct intercellular communication, and release of MVs for indirect intercellular communication (70–73). Both of these means of communication, however, have the potential to transfer similar cargo, including proteins and miRNAs to target cells (74).

### 2.3 Immune response modulation by MSCs

Within their microenvironment, MSCs can be licensed, or educated, into anti-inflammatory cells (32, 75). While the process of MSC licensing is poorly defined, specific factors are known to be necessary – namely IFNγ, in the presence of other pro-inflammatory cytokines (e.g. TNF-α, IL-1α, IL-1β) (76–78).

The immunomodulatory properties of MSCs were not well understood until recent studies showed that MSCs could impair the function of both innate and adaptive immune system cell proliferation (79, 80). These studies paved the way for scientists to shift their focus from the MSCs’ multiple-lineage and regenerative properties toward understanding the immune regulator capacity of these cells. MSCs regulate the immune system response through the release of MVs and soluble factors that impact the ability of the innate and adaptive immune cells such as myeloid DC and T-cells to respond to an infection (76, 81, 82). MSCs impair DC function by preventing their transition from immature to mature DCs, thus preventing presentation of antigen to naïve T-lymphocytes and decreased release of pro-inflammatory cytokines (e.g. TNF-α) (82, 83). MSCs also decrease NK cytotoxicity through decreased IFNγ release and decreased NK proliferation, in part through downregulating expression of NKp30 and NKG2D in NK cells (76, 84). MSCs further regulate the immune system by impairing the inflammatory response induced by the cells of the adaptive immune system. For example, MSCs inhibit T-lymphocyte differentiation by increasing IL-4 and decreasing IFNγ secretion (76). The decrease of pro-inflammatory cytokines allows T-lymphocytes to differentiate toward anti-inflammatory T_h_2 rather than pro-inflammatory T_h_1 phenotype (43, 76, 85). MSCs also act on macrophages by polarization from pro-inflammatory M1 to anti-inflammatory M2 phenotype (64, 86, 87). These findings have relevance to MSC function in the uterus, as MSCs isolated from human endometrium and menstrual fluid have immunomodulatory properties (88). Thus, it has been postulated, although unproven, that similar interactions between MSCs and uterine immune cells (e.g. uterine NK (uNK) cells and macrophages) play a role in promoting the immune microenvironment required for endometrial regeneration (89).

### 2.4 Regenerative potential of MSCs

MSCs, in addition to their crucial role in immune modulation, also play an essential role in maintaining host tissues’ homeostasis by replacing dead and dysfunctional tissue. The capacity of MSCs to sustain host homeostasis through the repair and replacement of dead and dysfunctional cells is predominantly attributed to their secretome, as discussed above. The effectiveness of MSC response to inflammation or injury is conditioned by their ability to home to the site of insult or injury. However, once MSCs are recruited to the site of inflammation, they can directly or indirectly interact with the affected tissue by GJIC or paracrine factors, as described earlier. Local inflammatory cytokines (e.g. TNF-α, IFNγ) signal MSCs to release immunomodulatory, pro-angiogenic, regenerative, and neuroprotective factors including TGFβ1, VEGF, HGF, SDF-1, IGF-1, and angi-1 (43, 62, 90, 91). These effects can be accomplished via the MSCs’ endogenous cargo or can be engineered to enhance these effects (21, 25, 92, 93). It is worth noting that MSCs are also being investigated as a drug delivery tool for gene therapy associated with these and other functions (73, 94–96). The self-renewing capacity of MSCs permits the MSCs to act as self-maintaining drug delivery vehicles at a site of inflammation so long as the microenvironment remains permissive to the MSCs (43, 97, 98).

Although research to date predominantly attributes MSCs’ effects to their secretome rather than cellular replacement, that is not to say that such function is not possible. MSCs have been found to generate osteocytes, adipocytes, chondrocytes, myocytes, and functional neurons *in vitro* (21, 22, 99–101). Numerous groups are investigating ways to accomplish similar direct reprogramming of MSCs and other cells *in vivo*, for example differentiating fibroblasts into neurons (102). With respect to endometrial regeneration, though paracrine mechanisms predominate, human *in vitro* studies indicate that MSCs may contribute as cellular progenitors of human endometrial stromal fibroblasts and decidual cells (103, 104).

### 2.5 Endometrium as a model of wound healing - role of MSCs

Menstruation, defined as shedding of the superficial (i.e., functionalis) layer of the endometrium in the absence of embryo implantation, occurs in only a restricted number of mammalian species: humans and certain non-human primates, the spiny mouse, several bat species, and elephant shrews (105). The human endometrium rapidly regenerates an entire tissue layer post menstruation, exhibiting multiple parallels with processes required for cutaneous wound healing (e.g. inflammation, tissue remodeling, fibroblast migration, and re-epithelialization) (106). Indeed, menstruation may be considered a model of tissue “self-damage” (107) followed by rapid, repeated regeneration without fibrosis in healthy women. The remarkable ability of the healthy endometrium to regenerate without fibrosis and scarring therefore lends itself as a unique model of scar-free wound healing (108).

In general, scar formation in repair of damaged tissues (e.g. skin) involves transformation of stromal cells into myofibroblasts, a key mechanism underlying fibrosis and scar formation. In contrast, post-menstrual repair of the endometrial “wound” occurs via tissue-specific mechanisms that prevent fibrosis and result in an entirely renewed and scarless functionalis layer. These mechanisms include: 1) unique ECM interactions mediating cell migration and re-epithelialization of the endometrium (109), and 2) soluble factors secreted *in utero* during menstruation which prevent transformation of endometrial stromal cells into myofibroblasts (107). Re: the latter mechanism, whether MSCs are one source of these soluble factors responsible for scar-free, post-menstrual cyclic endometrial repair in healthy women is not definitively known. Indeed, the endometrium itself, as well as menstrual fluid, are established sources of MSCs, among other stem/progenitor cell types, which secrete bioactive soluble and insoluble factors for potential clinical application in cutaneous wound healing [(reviewed in (88)]. MSCs, including those isolated from human endometrium, are thus an emerging therapeutic agent for addressing the inflammation, proliferation, and remodeling aspects of cutaneous wound healing – including reduced scar formation (88, 110–113). An expanding body of literature now demonstrates similar MSC and MSC-Exos-enhanced mechanisms in the endometrium, modulating inflammation, angiogenesis, and tissue remodeling. For these reasons, MSCs are under investigation in the treatment of disorders of endometrial regeneration, as seen in women with Asherman’s syndrome and thin endometrium refractory to exogenous estrogen. The following sections will expand on current research evaluating therapeutic value of MSCs on endometrial repair.

## 3 MSCs for treatment of thin endometrium - rodent studies

The particular ability of MSCs to cross allogeneic barriers has permitted delivery of human MSCs to rodents to study effects on endometrial regeneration and receptivity. Thus, an ever-growing number of studies have been conducted in rodents to explore the efficacy of MSCs, isolated from various rodent and human tissue sources, in treating thin endometrium. In the section below, we summarize a number of recent and pertinent studies which demonstrate various endometrial injury models, MSC sources, modes of delivery, and morphologic and functional outcomes of interest. As noted earlier, limited engraftment in recipient tissues and short retention time are major obstacles in MSC-based therapies. Several methods of MSC delivery have thus been tested in rodent models, including systemic delivery by tail vein injection, local injection directly into the uterus, or administration via a matrix system. These studies, summarized in **Table 1**, largely utilize three models to simulate endometrial injury (although other models have been described); injection of ethanol to achieve a thin endometrium, and mechanical or electrocoagulation injury of the endometrial surface to achieve the fibrosis typically seen in women with Asherman’s syndrome.

**Table 1:** MSC effects on thin endometrium: rodent studies.

Mode of Delivery	MSC Type	Species of MSC Origin/Recipient Species	Outcomes	Reference
Systemic injection	BM-MSC	Rat/rat	-↑endometrial thickness -↑anti-inflammatory cytokines -↓pro-inflammatory cytokines	Jing et al. (114)
Systemic injection	BM-MSC	Rat/rat	-↑endometrial thickness, gland, and vessel number -↑expression of HOXA10 and LIF	Xia et al. (115)
Systemic injection	BM-MSC	Rat/rat	-↓fibrosis -↑pregnancy rate	Gao et al. (116)
Systemic injection	BM-MSC	Rat/rat	-↑endometrial thickness, gland number -↓fibrosis	Xiao et al. (117)
BM-MSCs administered systemically, MSC-EVs administered locally	BM-MSC +MSC-EVs	Human/rat	-↑endometrial thickness, gland number, angiogenesis -↓fibrosis -↑endometrial receptivity -↑in cumulative number of pups	Mansouri-Kivaj et al. (118)
Systemic injection	MenSC	Human/rat	-↑endometrial thickness, vessel density -↑fertility	Zhang et al. (119)
Systemic injection	UC-MSC	Human/rat	-↑ESC proliferation -↑endometrial thickness, gland number -↑implantation sites -↓fibrosis	Zhang et al. (120)
Local administration	BM-MSC	Rat/rat	-↑endometrial thickness, gland number -↑endometrial receptivity markers -↑anti-inflammatory cytokines -↓pro-inflammatory cytokines	Zhao et al. (121)
Local administration	BM-MSC	Rat/rat	-↑endometrial thickness -↓fibrosis -↑endometrial receptivity markers -↑ fetal number	Wang et al. (122)
Local (myometrial) injection	AMSC	Human/rats	-↑endometrial thickness, gland number -↑angiogenic and anti-inflammatory cytokines -↓pro-inflammatory and pro-fibrotic cytokines	Gan et al. (123)
Local administration	ASC	Rat/rat	-↑endometrial thickness, gland number -↑receptivity markers, ERα, PR -↑angiogenesis -↑fertility	Shao et al. (124)
Local administration	BM-MSC-exos	Human/rat	-↑endometrial thickness, gland number -↓fibrosis -↑epithelial proliferation -↑stromal cell migration	Tan et al. (125)
Local administration	UMSC/UMSC-exos	Rat/rat	-↑proliferation and vascularization - ↓fibrosis (UMSC-exos only)	Saribas et al. (126)
Local administration	UC-MSC/UC-MSC-exos	Human/rat	-↑endometrial thickness, gland number, vessel density -↑LIF -↓fibrosis -↑pregnancy rates -↑HESC migration and proliferation *in vitro*	Zhang et al. (127)
Systemic injection and local administration	BM-MSC	Rat/rat	-↓fibrosis -↑gland number	Wang et al. (128)
Systemic injection vs. local administration	BM-MSC	Rat/rat	-↑endometrial thickness, vessel, and gland number -↓fibrosis -↑receptivity markers (systemic>local)	Guo et al. (129)
Local administration on day 1, followed by 1-3 systemic injections	UC-MSC	Human/rat	-↑gland and blood vessel density -↑implantation rates -↓fibrosis	Zhang et al. (130)
Systemic and local administration	UC-MSC-EVs	Human/rat	-↓fibrosis, pro-inflammatory cytokines -↑gland number -↑VEGF	Ebrahim et al. (131)
Systemic vs. local administration	BM-MSC or ASC	Rat/rat	-Local ASC administration provided greatest improvement in endometrial thickness and fibrosis	Monsef et al. (132)
Matrix (collagen scaffold)	BM-MSC	Rat/rat	-↑endometrial thickness, neovascularization -↑pregnancy rate	Ding et al. (133)
Matrix (hyaluronic acid)	HP-MSC	Human/rat	-↑endometrial thickness, gland number -↓ fibrosis -↑implantation sites	Lin et al. (134)
Matrix (acellular amniotic matrix)	UC-MSC	Human/rat	-↑endometrial thickness, gland number, anti-inflammatory cytokines -↓pro-inflammatory cytokines	Wang et al. (135)
Matrix (Matrigel microspheres)	UC-MSC	Human/rat	-↑endometrial thickness, gland number, blood vessel density -↑pregnancy rate	Xu et al. (136)
Matrix (PF-127)	UC-MSC	Human/rat	-↑endometrial thickness, gland number, angiogenesis	Zhou et al. (137)
Matrix (PPCNg)	AMSC	Human/rat	-↑endometrial thickness, gland number -↓fibrosis -↑pregnancy rate	Huang et al. (138)
Matrix (hyaluronic acid)	BM-MSC secretome	Human/rat	-↑endometrial thickness, gland number -↑implantation sites	Liu et al. (139)
Matrix (collagen scaffold)	UC-MSC-exos	Human/rat	-↑endometrial thickness, gland number -↑implantation sites -↓fibrosis -↑macrophage M2 (anti-inflammatory) phenotype	Xin et al. (140)

AAM, acellular amniotic matrix; AMSC, amniotic mesenchymal stem cell; ASC, adipose-derived mesenchymal stem cell; BM-MSC, bone marrow-derived mesenchymal stem cell; CS, collagen scaffold; eMSC, endometrial-derived mesenchymal stem cell; ESC, endometrial stromal cell; EVs, extracellular vesicles; Exos, exosomes; HA, hyaluronic acid; HESC, human endometrial stromal cell; HOXA10, Homeobox A10; HP-MSC, human placenta derived mesenchymal stem cell; LIF, leukemia inhibitory factor; Men-SC, menstrual blood-derived mesenchymal stem cell; MSC-EVs, mesenchymal stem cells-extracellular vesicles; PF-127, Pluronic F127; PPCNg, polyethylene glycol citrate-co-N-isopropylacrylamide mixed with gelatin; UC-MSC, umbilical cord-derived mesenchymal stem cell; UMSC, uterine MSC; UMSC-exo, uterine MSC-derived exosomes; VEGF, vascular endothelial growth factor ↑, increased; ↓, decreased.

### 3.1 Systemic MSC delivery

The earliest rodent studies to determine the effect of MSCs on endometrial regeneration were performed with systemic delivery of MSCs after inducing endometrial injury. These studies, summarized below, utilized MSCs from various sources and largely demonstrated improvement in endometrial thickness, gland number, and angiogenesis, as well as decreased fibrosis. Studies differ in terms of time point(s) studied post injury tracking of MSCs to the uterus, and assessment of fertility; those that perform mating studies largely demonstrated improvement in fertility in animals receiving systemically delivered MSCs.

In one of the earliest studies using a rat model of ethanol-induced endometrial injury (114), recipients of bone marrow-derived mesenchymal stem cells (BM-MSCs) post injury had a significantly thicker endometrium after 3 estrous cycles than saline controls. BM-MSC recipients also demonstrated increased expression of endometrial receptivity markers integrin αvβ3 and leukemia inhibiting factor (LIF), increased expression of endometrial bFGF and IL-6 mRNA (anti-inflammatory cytokines), and decreased expression of TNF-α, IL-1β mRNA (pro-inflammatory cytokines), indicating immunomodulation as one likely mechanism underlying reparative responses post endometrial injury. Subsequent studies found promising results after systemic BM-MSC delivery in terms of homing of cells to the uterus and improvements in fibrosis and endometrial morphology (115, 116). Mating studies demonstrated improved fertility in BM-MSC recipients, though not restored to that of uninjured controls (116). Interestingly, although BM-MSCs were localized in the endometrium of recipients up to 3 estrous cycles (115) or 3 weeks (116) post BM-MSC injection, none were noted in pregnant uteri of BM-MSC recipients (116), highlighting functional improvements despite short-lived retention of cells in the target tissue.

Given the well-established role of MSC extracellular vesicles (EVs) in mediating tissue repair, recent studies have characterized the role of BM-MSC-EVs in endometrial repair (117, 118). Xiao et al. (117) demonstrated an exosome-mediated repair process in a rat model of Asherman syndrome; improvements in morphology (decreased fibrosis, increased endometrial thickness and gland number) in BM-MSC recipients were mediated by exosomal miR-340. In a subsequent study, direct comparison between recipients of BM-MSCs vs. BM-MSC-EVs revealed similar improvements in endometrial thickness, gland numbers, and fibrosis, however improved fertility (number of deliveries and cumulative number of pups) was only noted in BM-MSC recipients (118). Thus, though EVs have emerged as a promising “off-the-shelf” cell-free therapy, further investigation is warranted to produce a dose and subpopulation of EVs that will elicit functional improvement comparable to the MSCs from which they were derived.

Studies using alternative sources of MSCs also yield promising results in rodent endometrial injury models. In a study exploring the therapeutic potential of human menstrual blood-derived MSCs (MenSCs), Zhang et al. demonstrated short term retention (up to 7d) of human MSCs in mouse recipient tissues, as well as increased endometrial thickness and microvessel density relative to saline controls (119). Improved fertility was noted with increased conception rates as well as a larger number of embryos. More recently, Zhang et al. employed human umbilical cord-derived MSC (UC-MSCs) as an alternative MSC source (120) and similarly found significant increases in endometrial thickness, gland number, and implantation sites, although not restored to that of uninjured controls. Complementary *in vitro* studies demonstrated the capacity of UC-MSCs to migrate toward injured rat endometrial stromal cells (ESCs) and enhance ESC proliferation (120), while bioinformatics analyses noted reconstruction of ECM, regulation of inflammatory molecules, cell proliferation and apoptosis as pathways associated with UC-MSC-mediated repair processes. All well-established properties of MSCs, these studies elucidated candidate mechanisms underlying UC-MSC-induced endometrial repair.

### 3.2 Local MSC delivery

Not long after the earliest studies demonstrating reparative effects of BM-MSCs delivered via tail vein (114), subsequent studies investigated the effect of local intrauterine MSC delivery on endometrial repair in endometrial injury models. Using various sources of MSCs, these studies demonstrated many of the same improvements after local transplantation that were seen with systemic administration, including increased endometrial thickness, gland number, vessel density, decreased fibrosis, and improved fertility.

In rat models of endometrial injury, two studies investigating effects of local intrauterine BM-MSC delivery demonstrated significantly increased endometrial thickness and higher expression of receptivity markers integrin αvβ3 and LIF in BM-MSC recipients (121, 122). Zhao et al. (121) also found downregulated expression of proinflammatory cytokines TNF-α and IL-1β, and upregulated anti-inflammatory cytokines bFGF and IL-6 in BM-MSC recipients, suggesting immunomodulatory mechanisms similar to those in rats treated systemically with MSCs. Mating studies by Wang et al. (122) demonstrated a significantly higher number of fetuses in the injured horn of BM-MSC recipients than in control animals receiving PBS. These studies provided support for local intrauterine BM-MSC administration to promote endometrial regeneration, potentially via immunomodulatory effects, and restore fertility after endometrial injury.

Looking at alternative MSC sources, Gan et al. performed one of the few studies to date exploring the use of human amniotic mesenchymal stromal cell (AMSC) transplantation in a rodent model of Asherman syndrome (123). In addition to improved thickness and gland numbers, the endometrium of AMSC recipients had significantly decreased expression of pro-inflammatory (e.g. TNF-α) and pro-fibrotic (TGF-β and COL1A1) cytokines, and increased expression of anti-inflammatory and angiogenic cytokines. This was the first study to demonstrate a role for AMSCs in endometrial regeneration; however, only short-term effects at one week post-transplant were studied. Fertility studies were not performed, thus their overall efficacy compared to more frequently tested sources of MSCs remains to be established.

More recently, Shao et al. explored the therapeutic potential of rat adipose-derived stem cells (ASCs) in a rat model of ethanol-induced endometrial injury (124). At 30 days, recipients of intrauterine ASCs had improved endometrial morphology including increased microvessel density, as well as increased expression of estrogen receptor (ER)α, ERβ, progesterone receptor (PR), LIF and integrin αvβ3 protein levels. Pregnancy rates were also improved in ASC-transplanted rats, indicating that ASC transplant could restore a functional endometrium to support embryo implantation.

To investigate the utility of a cell-free approach, several recent studies explored the effect of intrauterine MSC-exos delivery on endometrial repair (125, 126). In a mouse model of intrauterine adhesions, exosomes isolated from BM-MSC (BM-MSC-exos) promoted endometrial repair by increasing proliferation of endometrial epithelial cells and migration of stromal cells. Antifibrotic effects appeared to be mediated by exosomal miR-29a, via reduction in αSMA, Collagen I, Smad2, and Smad3 expression in murine endometrial stromal fibroblasts. To determine efficacy of a uterine source of MSCs, Saribas et al. explored the effects of locally delivered uterine MSC (UMSC) and their exosomes (UMSC-exos) on rat endometrial regeneration (126). Isolated from whole newborn rat uteri, these MSCs cannot be assumed to be comparable to MSCs isolated from human endometrium or menstrual fluid. Interestingly, although vessel density and cellular proliferation were similarly improved in UMSC and UMSC-exos recipients, fibrotic area was decreased only in uteri of the UMSC-exos group. A more recent study explored the utility of human umbilical cord MSCs and their exosomes (UC-MSC-exos) delivered via intrauterine infusion in a rat model of endometrial injury (127). In addition to morphologic improvements, pregnancy rates were significantly improved in the UC-MSC (60%) and UC-MSC-exos groups (80%), compared with zero pregnancies in untreated controls. UC-MSC and UC-MSC-exos inhibited TGF-β_1_/SMAD 2/3 (profibrotic) signaling in human endometrial stromal cells (HESCs) and upregulated HESC migration and proliferation *in vitro*, providing mechanistic insights into the functional improvements seen in the rat *in vivo* studies. Taken together, these studies elucidated multiple mechanisms by which local administration of MSC-exos could mediate endometrial repair after injury.

### 3.3 Systemic and local delivery, compared or combined

Though MSCs administered either systemically or locally have demonstrated favorable effects on endometrial repair in rodent models, few have directly compared the efficacy of systemic vs. local intrauterine administration. One comparison of rat BM-MSCs administered either systemically or locally in an intrauterine adhesion model (128) demonstrated significant and similar improvement in fibrosis and gland numbers in both the systemic and local treatment groups when compared to PBS-treated controls. A more recent study directly compared engraftment, cell retention, and therapeutic efficacy of rat BM-MSCs in a model of ethanol-induced injury, delivered either systemically or intrauterine (129). With either delivery method, BM-MSCs were localized to the endometrial stroma rather than epithelium and were equally effective in improving endometrial thickness, vessel, and gland density, and reducing fibrosis. However, the authors concluded intra-arterial delivery to be superior, given longer retention time and higher expression of VEGF and integrin αvβ3 protein in the endometrium. Fertility studies were not performed.

Zhang et al. utilized a combined systemic and local approach in a rat model, delivering human UC-MSCs locally at the time of endometrial injury, followed by 1-3 systemic injections of UC-MSCs (130). Endometrial morphology and fibrosis significantly improved in all UC-MSC-treated groups. Mating studies demonstrated the highest number of implantation sites in those receiving three systemic MSC injections in addition to intrauterine MSCs, though none recovered fertility to that of uninjured controls. An alternate model, taking a cell-free approach, tested the efficacy of a combined delivery system using locally delivered UC-MSC-extracellular vesicles (EVs) administered in the uterus on the day of endometrial injury and intraperitoneally 3 times at 5 day intervals with or without oral estrogen (131). Uteri of animals receiving UC-MSC-EVs with or without estrogen therapy exhibited many of the improvements noted in other studies (improvements in fibrosis, gland number and VEGF expression, significant decrease in expression of proinflammatory cytokines) relative to untreated animals or those receiving estrogen alone. Unfortunately, this study did not compare UC-MSCs alone to UC-MSC-EVs, and fertility after treatment was not tested.

A majority of the studies performed explore only 1-2 methods of delivery or one source of MSCs; however, Monsef et al. performed a unique study comparing administration of MSCs locally and systemically, as well as the performance of MSCs from either rat bone marrow or rat adipose tissue (132). Although MSC delivery by either method and from either source resulted in significant improvement in endometrial thickness after injury, local transplantation of both types of MSCs appeared to be more effective than systemic administration of ASCs, and systemic BM-MSCs were more effective than systemic ASCs in improving endometrial thickness. Systemic BM-MSC delivery, however, resulted in the fewest MSC visualized in the endometrium relative to other MSC groups. Both routes of delivery significantly increased endometrial VEGF expression and decreased collagen deposition, with the greatest improvement in fibrosis seen in animals receiving local ASC delivery, leading the investigators to conclude that ASCs may be a superior option for endometrial repair than BM-MSCs. Additional comparative studies, including assessment of fertility in MSC recipients, are needed to validate these findings.

### 3.4 MSC delivery by matrix

Given limited retention of MSCs in the endometrium after systemic or local delivery, a number of studies have investigated novel delivery systems to optimize delivery of MSCs (or their secretome), promote cell retention in the endometrium, and improve the effects on endometrial repair. A variety of matrices have been tested in combination with intrauterine delivery of MSCs derived from various tissues. Some studies utilizing these models demonstrate improved retention of cells in the uterus as well as improved fertility; all demonstrate positive effects on endometrial morphology after injury. However, in part due to heterogeneity in study design, only certain studies demonstrate superiority of the matrix over delivery of MSCs alone in promoting endometrial repair after injury.

In one of the initial studies evaluating matrix-based MSC delivery in a rat model of endometrial injury, Ding et al. explored the regenerative effects of rat BM-MSCs loaded on a scaffold of collagen, a basic structure of extracellular matrix and a widely used biomaterial in tissue regeneration (133). After 90d, animals receiving collagen/BM-MSCs had significantly thicker endometrium and greater degree of neovascularization relative to animals receiving either PBS or collagen alone. Mating studies demonstrated a higher pregnancy rate in collagen/BM-MSC recipients relative to collagen and untreated controls. However, lack of a BM-MSC treatment group precluded evaluation of potential benefits of collagen/BM-MSCs above BM-MSCs alone.

Multiple studies have tested novel matrix delivery systems for MSCs isolated from human pregnancy tissues (e.g. umbilical cord, placenta, amnion) (134–138), given their low immunogenicity and ease in collection at delivery. Lin et al. evaluated MSCs isolated from human placenta (HP-MSC) encapsulated in hyaluronic acid (HA) hydrogel for intrauterine delivery in a mouse model of endometrial injury (134). HP-MSC-HA complex significantly increased endometrial thickness and gland numbers, decreased fibrosis, and promoted cellular proliferation, though not significantly above that of HP-MSC alone. Further, despite prolonged MSC retention time in the HP-MSC-HA group, mating studies demonstrated a significant increase in implantation sites in recipients of HP-MSC with or without HA encapsulation; thus, from a functional standpoint, the superiority of administering MSCs within this matrix delivery system remained unproven.

In another study of novel delivery systems, Wang et al. investigated the efficacy of seeding human UC-MSCs on human acellular amniotic matrix (AAM) in a rat model (135). After 3 estrous cycles, endometrial thickness and gland numbers were significantly improved in the UC-MSC-AAM group relative to the AAM-treated group. Similar to findings from previous studies, UC-MSC-AAM delivery was also associated with upregulation of anti-inflammatory cytokines (IL-4, IL-10) and downregulation of pro-inflammatory cytokines (IL-2, TNFα, IFNγ) in the endometrium. Despite these effects, mating studies indicated zero pregnancies in rats transplanted with UC-MSC-AAM or AAM alone, ultimately raising doubt regarding any functional improvements afforded by this transplant method.

Xu et al. (136) noted limitations of thicker scaffolds, including restriction of nutrient diffusion, which can cause low viability and decreased colonization of MSCs *in vivo*. They thus tested the use of MSC-laden Matrigel microspheres in a rat uterine injury model, to improve nutrient diffusion and to facilitate MSC encapsulation and ease in transplant via injection (136). Rats receiving UC-MSC-laden Matrigel microspheres exhibited a significant increase in endometrial thickness, gland number, blood vessel density, and pregnancy rates relative to a control group receiving Matrigel alone. Though promising for a novel and less invasive MSC delivery system, lack of a UC-MSC treatment group again precluded a direct comparison of UC-MSCs delivered via this matrix delivery system above that of MSCs alone.

Thermosensitive biomaterials have gained attention for MSC delivery, transitioning from an easily injected liquid to a biodegradable hydrogel when exposed to body temperature. Zhou et al. tested a thermosensitive biodegradable hydrogel FDA-approved for clinical use, Pluronic F127 (PF-127), to deliver UC-MSCs into the uterus after ethanol-induced injury (137). Recipients of PF-127-encapsulated UC-MSCs had significantly increased endometrial gland numbers and angiogenesis relative to saline controls; these effects were not seen after delivery of UC-MSCs nor PF-127 alone. Huang et al. also explored use of a thermoresponsive biomaterial, polyethylene glycol citrate-co-N-isopropylacrylamide (PPCN) mixed with gelatin (PPCNg) to improve cell adhesion and survival of amniotic MSCs (AMSCs) (138). Endometrial thickness and gland numbers were found to be highest, and fibrosis decreased, in the AMSC-PPCNg group relative to AMSC or PPCNg only. Most notably, rats receiving AMSC-PPCNg treatment had the highest pregnancy rate (100%), as compared with 75% and 25% in the AMSC and PPCNg only groups, respectively. In both of these studies, retention of MSCs in the uterus was prolonged when delivered in a thermosensitive matrix; these studies provided compelling evidence supporting a novel delivery matrix for MSCs, warranting additional studies to determine safety and efficacy.

Given the limitations of cell-based MSC therapies (e.g. potential risk of tumor formation, low engraftment rates, storage and transportation logistics), and relative benefits of matrix-based delivery systems, a few studies have evaluated therapeutic effects of the MSC secretome delivered via matrix. Liu et al. (139), for example, evaluated the therapeutic effect of BM-MSC-secretome delivered via hyaluronic acid (HA) matrix in a rat model of endometrial injury. The HA+MSC-secretome group showed a significant improvement in endometrial thickness and gland number, and significant increase in number of implantation sites, in comparison to control and MSC-secretome only groups. *In vitro* studies demonstrated ability of the BM-MSC-secretome to promote angiogenesis, as well as proliferation and migration of human endometrial epithelial cells, providing mechanisms underlying the improvements seen in their *in vivo* studies. In another matrix-based model, Xin et al. employed the use of a collagen scaffold for intrauterine UC-MSC-exos delivery after uterine injury in rats (140). UC-MSC-exos (delivered with or without the scaffold) significantly increased gland number and endometrial thickness, reduced fibrosis, and improved fertility, with a significantly higher number of implantation sites seen in UC-MSC-exos recipients. Consistent with known functions of MSC-exos, recipients of UC-MSC-exos exhibited polarization of endometrial macrophages to the anti-inflammatory M2 phenotype, likely mediated in part by exosomal miR-223-3p, supporting the role of MSC-exos cargoes in promoting endometrial repair. Collectively, these studies incorporated favorable aspects of two MSC-based delivery methods (cell-free/secretome-based combined with matrix for sustained release) to ameliorate endometrial morphology and function after injury.

Taken together, a growing number of rodent studies have demonstrated efficacy of several MSC sources and administration methods for the treatment of thin endometrium and/or intrauterine adhesions. The most widely tested of these include BM-MSCs and UC-MSCs: all show the ability in rodent models to improve endometrial regeneration, and multiple demonstrate improved fertility. Direct comparison between sources are lacking; only one study performed a direct comparison of efficacy between two MSC sources (BM-MSCs and ASCs), and concluded that ASCs may be a superior option for endometrial repair than BM-MSCs (132). Very few studies compare mode of delivery (systemic vs. local administration) and yielded mixed results. Of the various MSC delivery vehicles evaluated, only a few directly compare efficacy of MSCs (or MSC secretome) delivered in a matrix to those delivered alone; in those studies, the data indicate these delivery complexes may be beneficial in prolonging tissue retention of MSCs and improving endometrial regeneration.

These rodent studies have overall improved our understanding of the mechanisms by which MSCs support endometrial regeneration, and many have laid the groundwork for human studies. Both the human and rodent endometrium undergo cyclic remodeling which is highly dependent on ovarian sex steroids and a functional hypothalamic-pituitary-ovarian axis (141). Over the course of either the rodent estrous cycle (4-5 days) or human menstrual cycle (28 days), the endometrium undergoes distinct changes including stromal and epithelial proliferation, apoptosis, extracellular matrix remodeling and leukocyte infiltration, under the influence of estradiol and progesterone (142, 143). Thus, many parallels exist between rodents and women, but the lack of menstruation in rodents renders this species as an imperfect model, albeit convenient and easy to manipulate, for *in vivo* studies of endometrial regeneration and repair. It is important to keep these similarities and differences in mind when extrapolating the results seen in rodents to findings from human studies. *In vitro* studies in human tissues, summarized in the section below and **Table 2**, have provided valuable additional insights into MSC-mediated endometrial repair mechanisms.

**Table 2:** MSCs promote endometrial repair mechanisms: Human *in vitro* studies.

MSC Type	Target cells studied	Model of exposure	Findings	Reference
UC-MSC	HESCs	-Collagen scaffold -Transwell co-culture	-↑ proliferation -↓ apoptosis -↑VEGF-α, TGF-β, IGF-1	Xin et al. (140)
UC-MSC	HESCs injured with mifepristone	-treatment with UC-MSC-exos	-↑HESC survival and proliferation -↓ apoptosis via ↑ Bcl-2 and ↓ cleaved caspase-3	Wang et al. (144)
UC-MSC	HESCs injured with mifepristone	-treatment with UC-MSC-exos	-↓apoptosis via exosomal miR-7162-3p	Shi et al. (145)
UC-MSC	HESCs treated with TGFβ1 to induce fibrosis	-treatment with UC-MSC-exos	-exosomal miR-145-5p mediated reversal of fibrosis	Li et al. (146)
UC-MSC-exos	EECs (hypoxia-induced injury)	-treatment with UC-MSC-exos	-↑proliferation -↓hypoxia-induced apoptosis, migration, EMT -protective effects mediated by miR663-a/CDKN2A axis	Wang et al. (147)
BM-MSC and UC-MSC	HESCs	-transwell co-culture MSCs/HESCs	-↑CCL2, HGF -↑HESC proliferation, migration, invasion	Zhao et al. (148)
MenSC	EECs	-transwell co-culture MSCs/EECs	-↑ proliferation -↑ motility	Zhao et al. (149)
MenSC	HESCs injured with mifepristone	-transwell co-culture MSCs/HESCs	-↑ proliferation, migration and ↓ apoptosis via AKT and p38 MAPK pathways	Zhu et al. (150)
MenSC	HESCs	-HESCs cultured in MenSC conditioned medium	-attenuate fibrosis via secretory products of MenSCs (G-CSF)	Lin et al. (151)
MenSC	HESCs	-transwell co-culture MSCs/HESCs	-↓ myofibroblast markers -↑ HESC proliferation, migration	Zhu et al. (152)

ASC, adipose-derived mesenchymal stem cell; BM-MSC, bone marrow-derived mesenchymal stem cell; CS, collagen scaffold; eMSC, endometrial-derived mesenchymal stem cell; exos, exosomes; G-CSF, granulocyte-colony stimulating factor; HESC, human endometrial stromal cell; HGF, hepatocyte growth factor; IGF-1, insulin-like growth factor-1; MAPK, mitogen activated protein kinase; MenSC, menstrual blood-derived mesenchymal stem cell; TGF-β, transforming growth factor-β; UC-MSC, umbilical cord-derived mesenchymal stem cell; VEGF-α, vascular endothelial growth factor-α. ↑, increased; ↓, decreased.

## 4 MSCs to treat thin endometrium: human studies

### 4.1 Human *in vitro* studies

Among the first studies to explore paracrine mechanisms by which MSCs mediate human endometrial repair, Xin et al. used a collagen scaffold (CS) loaded with human UC-MSCs and studied the effect on human endometrial stromal cells (HESCs) (140). HESCs exposed to CS-UC-MSCs demonstrated significantly increased proliferative capacity and decreased apoptosis relative to controls, and secreted higher levels of VEGF-α, TGF-β, and insulin-like growth factor 1. These *in vitro* findings were further validated by the same group in a rat model of uterine injury, in which the application of CS-UC-MSCs into the uterus after injury resulted in improved endometrial morphology and higher pregnancy rates. The endometrium of CS-UC-MSC rat recipients also exhibited higher expression of VEGF-α, TGF-β, and insulin-like growth factor 1, supporting MSC-mediated paracrine upregulation of these growth factors as a mechanism promoting morphologic and functional restoration of injured uteri.

As summarized earlier, MSC-derived exosomes contain cargo, including transcription factors and abundant microRNAs (miRNAs), that regulate expression of related genes in recipient cells and promote repair and regeneration in damaged tissues (117). A number of recent human *in vitro* studies have thus explored mechanisms by which UC-MSC-exos promote survival and proliferation of HESCs, utilizing various *in vitro* models of HESC injury (144). Exposure to UC-MSC-exos significantly increased HESC proliferation and decreased apoptosis; mediated via activated PTEN/Akt signaling in mifepristone-injured HESCs, upregulated HESC expression of Bcl-2 (an anti-apoptotic protein) and downregulated cleaved Caspase-3, a marker of apoptotic injury (144). In a similar model, Shi et al. (145) identified miR-7162-3p as an effector of UC-MSC-exosome endometrial repair. Direct binding of miR-7162-3p to apolipoprotein 6 (APOL6), a programmed cell death gene in target HESCs, protected against mifepristone-induced injury. Li et al. also investigated therapeutic potential of UC-MSC-exos in reversing TGFβ1-induced HESC fibrosis (146). Exposure of injured HESCs to UC-MSC-exos resulted in significant down regulation of fibrosis markers α-SMA1 and COL1A1 relative to controls. This finding was mediated at least in part by exosomal miR-145-5p, a negative regulator of the transcription factor ZEB2 which promotes epithelial-mesenchymal transition and fibroblast differentiation. With respect to endometrial epithelial cells (EECs), Wang et al. (147) demonstrated that uptake of UC-MSC-exos inhibited hypoxia-induced apoptosis, migration, reversed epithelial mesenchymal transition (EMT) of EECs, and upregulated EEC proliferation relative to untreated, injured controls. The protective effect of UC-MSC-exos appeared to be mediated by regulation of the miR663-a/CDKN2A axis, the latter an apoptosis regulatory gene. Collectively, these studies provided important insights into mechanisms by which miRNA cargo of UC-MSC-exos could support endometrial repair.

While the studies above largely focused on MSC-mediated mechanisms regulating HESC proliferation, survival, and anti-fibrotic mechanisms, our lab investigated whether the MSC secretome could enhance endometrial stromal cell motility functions, and whether MSCs isolated from either UC or BM would have similar effects (148). Exposure of HESCs to the secretome of either UC- or BM-MSC significantly and similarly increased HESC migration and invasion; although cellular proliferation was also significantly increased, the effect was modest and varied among MSC donors. Expression of CCL2 and HGF mRNA, genes associated with HESC motility, were increased in HESCs exposed to both UC- and BM-MSC, and exposure to recombinant CCL2 significantly increased HESC migration and invasion. These findings indicated both paracrine and autocrine mechanisms involved in MSC secretome-mediated motility, further supporting the potential to leverage the MSC secretome as a cell-free therapy to support endometrial regeneration/repair.

The human endometrium and menstrual fluid are now established sources of MSCs, and multiple *in vitro* studies have demonstrated the ability of menstrual blood MSCs (MenSCs) to affect phenotype and function of endometrial epithelial and stromal cells. Zhao et al. (149). demonstrated that exposure of EECs to MenSCs increased EEC proliferation and invasive capacity, and increased EEC expression of EGF, FGF, PDGF and MMP3 proteins; these cytokines and growth factors play important roles in cellular motility, proliferation, and tissue remodeling in the endometrium. With respect to effects on HESCs, in a model of mifepristone-induced injury, Zhu et al. demonstrated the ability of MenSCs to upregulate endometrial stromal cell proliferation, migration, and decrease apoptosis via activation of p38 MAPK and AKT signaling pathways (150). MenSCs and their secretory products (e.g., G-CSF) appear to attenuate endometrial fibrosis (151, 152); exposure of HESCs to either MenSCs or MenSC-conditioned medium resulted in downregulation of myofibroblast markers αSMA and collagen I and promoted HESC migration (152). Interestingly, as noted earlier, TGF-β is an inducer of fibrosis in the endometrium, and exposure to MenSCs attenuated TGF-β-mediated increase in profibrotic markers and myofibroblast activation, via activation of the Hippo/TAZ signaling pathway (152). Thus, although untested *in vivo*, these *in vitro* studies elucidated candidate MenSC-mediated mechanisms that promote scar-free endometrial repair in healthy tissue and identified potential molecular targets in the therapy of endometrial fibrosis/scarring.

### 4.2 Human trials

Santamaria et al. were one of the first groups to explore the use of bone marrow-derived stem cells (BMDSCs) in the uterus in a human trial (153). In a prospective, non-randomized study, 16 women with primary infertility and either Asherman’s syndrome (n=11) or endometrial atrophy (EA, n=5) received autologous CD133^+^ BMDSCs, delivered via intra-arterial catheterization of uterine spiral arterioles, followed by hormone replacement therapy. The endometrium of recipients was assessed prior to and at 3 and 6 months post treatment. Average endometrial thickness increased only modestly, from 4.3 mm to 6.7 mm post-treatment in women with Asherman’s syndrome; and from 4.2 mm to 5.7 mm in women with EA. Of 14 embryo transfers performed, 7 women became pregnant resulting in one live birth and one ongoing pregnancy; 3 patients conceived spontaneously at 2, 4, and 19 months after treatment resulting in one live birth and one ongoing pregnancy. No adverse events were noted with BMDSC treatment. It should be noted that the selection of CD133 as a marker indicates the use of a potentially heterogeneous stem/progenitor population not truly limited to MSCs. Although limited by lack of controls and unproven homing of BMDSCs to the uterus, this was a novel and important proof-of-concept pilot study setting the stage for subsequent trials in humans.

In a subsequent report by Singh et al., 25 women with infertility and Asherman’s syndrome (n=12) or thin endometrium (n=13) underwent transplant of autologous bone marrow-derived mononuclear stem cells (154). Cells were transplanted transmyometrially into the subendometrial zone, followed by 3-6 months of oral estrogen therapy. Of the 7 amenorrheic women, 6 resumed menses after treatment. Average endometrial thickness improved modestly from 3.3 to 5.1 mm at 3 months, without further improvement at 6 and 9 months. Eleven patients underwent IVF, of which only two conceived, resulting in one live birth and one ectopic pregnancy. Two women conceived naturally at 3 years post-transplant, resulting in one live birth each. The infusion of mononuclear cells includes a heterogenous population of hematopoietic and nonhematopoietic bone marrow-derived cells not limited to MSCs; further, lack of controls (and conception at several years post-transplant) precluded the ability to directly attribute pregnancies to the intervention.

In one of the earliest studies using a menstrual source of MSCs in the human uterus, Tan et al. studied the effects of autologous transplantation of MenSCs on endometrial regeneration (155). Unlike prior human trials, in this trial MSC phenotype was confirmed via expression of MSC markers. Seven women with Asherman’s syndrome underwent ultrasound-guided intrauterine transplant of MenSCs on day 16 of the menstrual cycle under ultrasound guidance, followed by oral estrogen for 21 days. Five of 7 patients achieved an endometrial thickness of 7-8 mm and underwent IVF with embryo transfer, of which two conceived. One patient naturally conceived 3.5 months after transplant. In a more recent study, Ma et al. also used MenSCs in 12 patients with refractory Asherman’s syndrome (156). These investigators also characterized MSC phenotype via cell surface marker expression and multilineage differentiation potential. MenSCs were transplanted into the basal layer of the endometrium, followed by 1-2 cycles of hormone therapy. Mean endometrial thickness was significantly improved at 14 days post-transplant, from 3.9 to 7.5 mm. The clinical pregnancy rate was 41.7% (5/12), 4 of which resulted from IVF/embryo transfer and 1 conceived spontaneously. Though promising, live births were not reported in either study, with lack of controls and small sample sizes limiting applicability of the results.

To explore an alternative MSC source, Sudoma et al. performed the first trial with adipose-derived stem cells (ASCs) in 25 women with thin endometrium and at least 3 prior IVF failures (157). MSC phenotype was confirmed via multilineage differentiation assays. Autologous ASCs were injected subendometrially in several locations under ultrasound guidance 3 times at 5–7-day intervals followed by oral estrogen. Endometrial thickness was measured in 3-10 natural cycles post-treatment, or if the patient was menopausal or had ovulatory dysfunction, after 3-10 artificial cycles. 19 women achieving endometrial thickness >7mm underwent embryo transfer. A total of 13 women achieved pregnancy: 11 after embryo transfer and 2 as a result of natural conception, resulting in a total of 9 live births. Compared to prior studies, this cohort of patients experienced the highest pregnancy and live birth rates, but again lacked controls.

Tersoglio et al. performed a study using autologous endometrial MSCs (eMSCs) in 29 patients with thin endometrium and recurrent implantation failure, defined as failed embryo implantation after at least 3 transfers of high-quality blastocysts (158). Autologous endometrial MSCs, characterized by expression of traditional MSC cell surface markers and eMSC marker SUSD2, were delivered transmyometrially after estrogen supplementation for 6-8 weeks. Average endometrial thickness increased from 5.2 mm pre-treatment to 9.9 mm. Of 29 women undergoing embryo transfer, the authors reported 23 clinical and 7 ongoing pregnancies, promising outcomes tempered by lack of controls and the major caveat that in all women, eMSCs were diluted in platelet-rich plasma, bringing into question the therapeutic role of eMSCs themselves.

Zhang et al. explored the use of UC-MSCs on endometrial regeneration in 16 infertile women with thin endometrium (< 5.5 mm) (159). UC-MSCs, confirmed by cell surface marker expression and multilineage differentiation potential, were loaded onto a collagen scaffold and transplanted hysteroscopically into the uterine cavity on the 7-12^th^ day of the menstrual cycle in 2 sequential cycles. Three months after transplantation, average endometrial thickness increased from 4.1 mm to 5.9 mm, with significant increase in CD34^+^ micro-vessel density, gland number, and expression of ERα, PR, and the proliferative marker Ki67. Of 15 women undergoing 22 frozen embryo transfer (FET) cycles, 3 conceived, 2 of which resulted in live birth; 1 patient had a naturally conceived pregnancy and live birth. In a similar trial, Cao et al. administered intrauterine UC-MSC on a collagen scaffold after hysteroscopic adhesiolysis in 26 women with infertility and intrauterine adhesions (160). Subjects received estradiol for 30 days followed by a single dose of 60 mg progesterone. At 3 months, 10/26 women were noted to have either absent or mild adhesions, with modest increases in average endometrial thickness from 4.5 mm to 5.7 mm and upregulated expression of ERα, proliferative marker Ki67, and angiogenic marker vWF. At 30 months, 10 subjects conceived a pregnancy, resulting in 8 live births, one ongoing pregnancy in third trimester, and one first trimester spontaneous abortion. No adverse events were reported in either trial. Though uncontrolled, these studies provided important safety data and insights into mechanisms by which intrauterine UC-MSC administration, via degraded collagen scaffold, could improve endometrial function.

Overall, pregnancies and live births in women with refractory Asherman’s syndrome and/or thin endometrium, coupled with a lack of treatment-related adverse events, generate enthusiasm for the development of novel MSC-based therapies to improve fertility in women suffering from these challenging conditions. However, major caveats exist, and proper randomized controlled trials are required before MSCs can be considered for clinical use. All of the above are uncontrolled studies with small sample sizes and heterogeneous clinical protocols; thus, none can definitively attribute clinical successes to MSC-based therapies. Heterogeneity among studies in transplanted cell types/cell populations raise uncertainty regarding the specific stem/progenitor cell types involved. Mechanisms responsible for clinical outcomes seen in humans, as well as the optimal mode of delivery, remain unknown. Finally, although MSCs from different tissues of origin are predicted to show slight differences in efficacy, and differing methods of MSC administration are expected to show differences in homing and target tissue residency (43) none of the reviewed trials compared different MSC sources or administration methods within the same cohort of patients. Thus, the optimal MSC source or administration method for ameliorating thin endometrium remains unknown.

## 5 Summary/future directions

**Figure 2:**
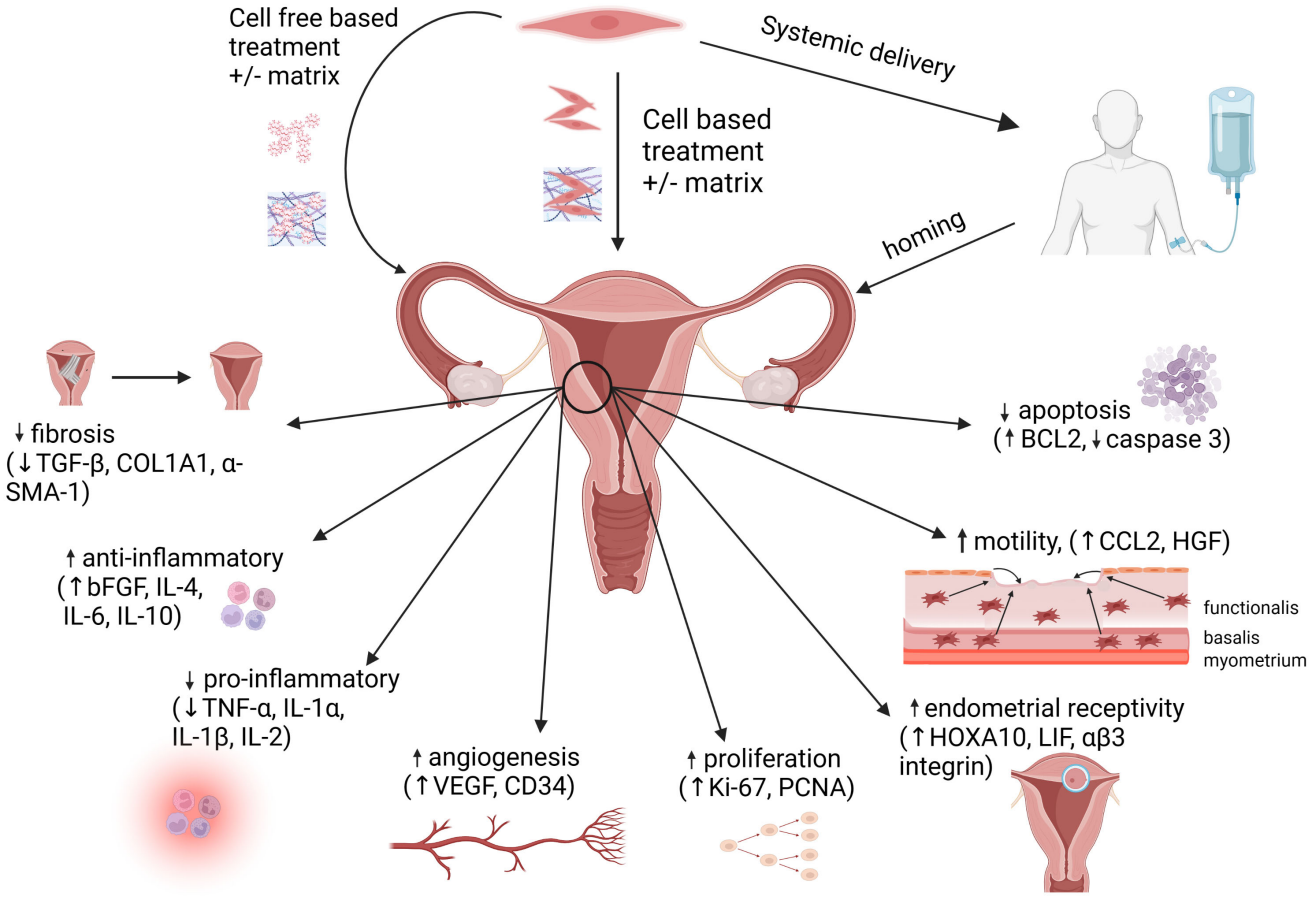
MSC administration methods and mechanisms of action. Mesenchymal stem cells (MSCs) can be delivered via direct administration (+/- matrix) into the uterus, or by systemic delivery, followed by homing of the MSCs to the endometrium. Alternatively, cell-free therapies utilizing the MSC secretome (+/- matrix) may be administered directly into the uterus. At the site of injury within the endometrium, MSC-based therapies promote regeneration via immunomodulation, increased angiogenesis, increased cellular proliferation and motility, decreased apoptosis and downregulation of pro-fibrotic pathways. Figure created with Biorender.com.

The studies described herein demonstrate an ever-expanding amount of work supporting the therapeutic potential of MSCs, derived from reproductive and non-reproductive tissues, in promoting endometrial regeneration and repair after injury. Rodent (*in vivo*) and human (*in vitro*) studies have elucidated mechanisms that are consistent with well-established functions of MSCs and their secretome: angiogenesis, amelioration of fibrosis, increased cellular proliferation and motility, and immunomodulation (**Figure 2**). Although MSCs and their secretory products hold great promise in treating infertility due to a thin or scarred endometrium, caveats remain. Effects seen in rodents may not fully extrapolate to humans. Results generated from human clinical studies to date are limited by small sample size, heterogeneity in protocols, inconsistency in MSC characterization, and critically, lack of controls. The use of cell-based vs. cell-free (secretome-based) therapies each have relative benefits and drawbacks, and the optimal mode of delivery into the human uterus (systemic, local +/-matrix-based) needs to be determined. Before MSCs can be applied for treatment in this context, their isolation and preparation for clinical use will need to adhere to current good manufacturing practice regulations to assure safety, standardization and reproducibility (161). Working to achieve this is a worthwhile endeavor, as endometrial factor infertility remains a rate-limiting step in achieving IVF success. If proven efficacious and safe, MSC-based therapies for thin endometrium will not only improve pregnancy rates; equally and vitally important, they will minimize the risk of placental dysfunction related to thin endometrium, thus setting the stage for healthier pregnancies and live births.

## Author contributions

MS-N: Writing – original draft, Data curation. YK: Writing – original draft, Data curation. LS: Writing – original draft, Writing – review & editing. OC: Writing – original draft, Data curation. SM: Writing – original draft, Writing – review & editing, Conceptualization, Data curation, Supervision.
